# Effects of Injury Registry Data on Policymaking, Hospitalizations, and Mortality: Systematic Review

**DOI:** 10.2196/67115

**Published:** 2025-09-10

**Authors:** Ana Cláudia Medeiros-de-Souza, Luana Emanuelly Sinhori Lopes, Tayna Felicissimo Gomes de Souza Bandeira, Lucas Reis Correia, Naiza Nayla Bandeira de Sá, Bruno Zocca de Oliveira

**Affiliations:** 1 Hospital Israelita Albert Einstein, 755 Comendador Elias Jafet Street, L1 Floor, Room 134, São Paulo, 05653-000, Brazil; 2 Department of Preventive Medicine, University of São Paulo, São Paulo, Brazil; 3 Secretariat of Health and Environmental Surveillance, Department of Surveillance of Noncommunicable Diseases and Health Promotion, Ministry of Health, Brasília, Brazil; 4 Faculty of Nutrition, Federal University of Pará, Belém, Brazil

**Keywords:** injury registry, trauma registry, policymaking, health policy, wounds and injuries, prevention and control, injury, trauma, Brazil, Newcastle-Ottawa Scale, PRISMA, systematic review, registry, registry data, injury outcome, hospitalization, mortality, morbidity, health surveillance, quality information, epidemiology, hospital care

## Abstract

**Background:**

The Brazilian project, launched in 2021, aims to establish a nationwide injury registry that systematically collects detailed information on incidents and individuals across the country, regardless of injury severity. The registry integrates information from prehospital and hospital care, various health systems lacking interoperability, and data from sectors such as firefighters and police. Its primary aim is to enhance health surveillance by providing timely, high-quality information that guides prevention strategies and informs policymaking. In addition, the project seeks to reduce morbidity and mortality associated with injuries.

**Objective:**

This study aimed to investigate the effects of injury registry data on policymaking, hospitalization rates or duration, and mortality.

**Methods:**

The systematic review followed PRISMA (Preferred Reporting Items for Systematic Reviews and Meta-Analyses) guidelines, with a protocol registered in PROSPERO (International Prospective Register of Systematic Reviews, CRD42023481528). A total of 5 databases were searched in November 2023, with an update conducted in March 2024, incorporating reference lists from the studies included. Two reviewers independently screened records, extracted data, and assessed methodological quality using the Newcastle-Ottawa Scale, resolving disagreements with a third reviewer. Studies were eligible if they reported results related to the implementation and use of injury or trauma registry data for at least one outcome of interest, while those based on other sources were excluded. Synthesis of findings was presented in tables, and the observed results were reported as number or percentage differences.

**Results:**

Out of 9100 studies retrieved, 3951 were excluded due to duplication, leaving 5149 for selection, with 15 full texts reviewed. Only 5 studies met the inclusion criteria, highlighting a notable scarcity of research on the effects or results of registry data on injury outcomes. It is important to note that the studies included reflect correlations rather than causalities, and there are currently no publications on impact. The findings suggest that injury and trauma registries have the potential to inform policymaking, which can lead to enhanced health outcomes. One study noted a 3-day reduction in intensive care unit stay (from 16 to 13 days; *P*<.05) and a 4% reduction in expected hospital mortality (from 17.5% to 21.5%) for patients with an Injury Severity Score ≥16, while another showed a 42% annual decrease in traffic injury hospital admissions (from 45 to 16). Significant methodological heterogeneity and the small number of studies limited the feasibility of a meta-analysis.

**Conclusions:**

Establishing an injury registry in Brazil presents a significant opportunity to enhance health outcomes through informed policymaking. While it is crucial to set appropriate expectations regarding its effects on morbidity and mortality, particularly concerning the causality and transportability of the findings to the Brazilian context, its role in facilitating preventive measures and improving surveillance capabilities remains valuable.

## Introduction

Injuries resulting from various incidents, such as accidents, falls, drownings, burns, poisonings, and acts of violence directed at oneself or others, represent a global concern. Annually, approximately 4.4 million deaths are attributed to injuries, constituting nearly 8% of all deaths. Unintentional causes account for 3.16 million fatalities, while acts of violence result in 1.25 million deaths. According to the World Health Organization, among individuals aged 5‐29 years old, 3 of the top 5 causes of death are injury-related: road traffic injuries, homicide, and suicide. In addition, falls lead to over 684,000 deaths each year, highlighting a growing and often overlooked public health issue [1].

In Brazil, injuries and violence have frequently ranked as the fourth leading cause of death. In 2022, the country recorded 152,945 fatalities attributed to injuries, which corresponds to approximately 75 deaths per 100,000 inhabitants, constituting nearly 10% of all deaths. Most of these fatalities were due to accidents (46.9%), with traffic accidents accounting for 22.8% and falls for 11.8%. Homicides represented 29.0% of the deaths, while suicides accounted for 10.8%. Studies highlight the significant impact of injuries on premature mortality and disability, particularly among young individuals aged 20‐39 years old and males, emphasizing the urgent need to prioritize this issue in the country [2,3].

Conversely, nonfatal injuries also pose a significant public health challenge. Every year, millions of people worldwide experience nonfatal injuries, resulting in visits to emergency departments or hospitalizations [1]. In Brazil, in 2022, the number of hospitalizations due to injuries was nearly 8.8 times higher than the number of deaths, totaling 1,351,021 hospitalizations, which translates to over 600 injury-related hospitalizations per 100,000 Brazilians. Falls were the most prevalent cause of these hospitalizations, accounting for 34.4%, followed by traffic accidents at 17.9% [4].

The National Policy for the Reduction of Morbidity and Mortality from Accidents and Violence was established in response to the challenge posed by injuries as a public health issue in Brazil [5]. This policy has led to several key initiatives, including the launch of the National Policy for Emergency Care in 2003 [6] and the proposal for creating the National Network for the Prevention of Violence and Health Promotion in 2004 [7]. Two years later, in 2006, the Violence and Accident Surveillance System in Sentinel Services was implemented [8]. In 2010, the Life in Traffic Project was initiated, emphasizing intersectoral coordination and data integration for traffic accident surveillance and intervention across 54 Brazilian municipalities [9,10]. Furthermore, beginning in 2011, interpersonal and self-inflicted violence were included in the list of diseases and conditions subject to compulsory notification in public and private health services in the country [11].

An injury registry is a systematic and standardized database that records detailed information about events and individuals who have sustained injuries, regardless of their severity or whether they result from accidents, falls, violence, or other causes. These databases can encompass a wide range of injuries, from minor ones that may not require hospitalization to more serious injuries that do. Traumatic injuries that require acute care delivered to hospitalized patients, typically resulting from high-impact events such as car accidents, severe falls, or gunshot wounds, are usually included in trauma registries. Both types of registries play important roles in injury prevention, research, and health care management. The primary distinction lies in the scope and severity of injuries each type of registry deals with. They allow policy makers to efficiently target resources and implement evidence-based interventions [12-15].

Recently, in 2021, a project was initiated under the Institutional Development Support Program of the Unified Health System (Programa de Apoio ao Desenvolvimento Institucional do Sistema Único de Saúde [PROADI-SUS]), a strategy of the Brazilian Ministry of Health, executed by the Hospital Israelita Albert Einstein, to establish a national injury registry. The primary aim of this initiative is to enhance health surveillance capacity by providing timely, high-quality information to guide the development and evaluation of prevention strategies and policymaking related to accidents and violence. To achieve this, the project focuses on improving data integration and quality from prehospital and hospital care, as well as from various existing health information systems that currently lack interoperability. In addition, it seeks to incorporate data from multiple sectors involved in injuries and violence, including firefighters and police. The objective is to consolidate information from diverse systems into an integrated database that will be centrally accessible to health managers and researchers throughout Brazil by 2026. Ultimately, these efforts are designed to reduce morbidity and mortality associated with injuries [16,17].

The success of trauma systems has been reported in various localities, with their effectiveness well-established [14,18-20]. Estimates indicate a 15% reduction in the odds of mortality [21,22]. Furthermore, the implementation of trauma registries has been well-documented, and these registries are consistently recognized as essential tools for improving trauma systems and policymaking [13,23]. However, a significant knowledge gap remains regarding the effects of injury registries that encompass all injuries, regardless of their severity or whether hospitalization is required [23].

Considering the current efforts to implement an injury registry in Brazil, there is an urgent need to establish an evidence base in this context. Therefore, this systematic review aimed to investigate the effects and results of injury registries on policymaking and injury outcomes, particularly in terms of reducing hospitalizations and mortality. Hence, the research question guiding this review is as follows: “What is the effect of implementing an injury registry on policymaking, hospitalization rates or duration, and mortality due to injuries?”.

## Methods

### Protocol

The development of this study followed the recommendations of the Preferred Reporting Items for Systematic Reviews and Meta-Analyses (PRISMA) statement, ensuring adherence to best practices in systematic review methodology [24,25]. The PRISMA 2020 checklist for a systematic review report is completed and provided in Checklist 1, while the checklist for the abstract is provided in Checklist 2. A protocol for this systematic review was registered in the PROSPERO (International Prospective Register of Systematic Reviews) database under registration number CRD42023481528. A comprehensive version of the protocol has been published [26].

### Eligibility Criteria

The eligibility criteria for this systematic review were formulated according to the PICOS structure (Population, Intervention, Comparison, Outcomes, and Study), as shown in Table 1. A search was conducted for articles reporting results or effects related to the implementation and use of data from injury registries, including trauma registries, for at least one of the outcomes described in Table 1. These registries were established within cities, states, provinces, countries, hospitals, or other well-defined administrative boundaries. Nonrandomized intervention studies were considered for inclusion, which involve those that cannot use randomization to allocate individuals or clusters of individuals into intervention groups, including quasi-experimental studies. Analytical observational studies, such as cohort studies and before-and-after studies, were also considered for inclusion. Only nonrandomized intervention studies and analytical observational studies could adequately address the research question that guided this systematic review, as a randomized controlled trial would not be feasible in this context.

**Table 1.** Eligibility criteria presented in the population, intervention, comparison, outcomes, and study structure.

Acronym	Description	Criteria
P	Population	Cities, states, provinces, countries, hospitals, or other well-defined administrative boundaries.^a^
I	Intervention	Implementation and use of injury registries, including trauma registries.
C	Comparison	No restrictions on comparators
O	Outcomes	Policymaking (changes and design, including preventive measures, health care improvements, surveillance actions, among others), hospitalization rates or duration, and mortality.
S	Study	Nonrandomized intervention studies or observational studies

a The population consists of the locations where injury registries have been implemented, with no restrictions on the patients served by health services regarding age, gender, or other attributes.

Studies were excluded if they were based on sources other than an injury or trauma registry. Articles without full-text availability were also excluded after attempts to contact the corresponding authors to request the full text. In addition, opinion articles, editorials, letters to the editor, conference abstracts, systematic or literature reviews, and any other studies that lacked empirical research regarding the effects of injury registries. The studies excluded were documented in an attached table within the systematic review. There were no restrictions on publication date, country, or language.

### Information Sources, Search, and Selection Process

The search was conducted in the MEDLINE databases via PubMed, Embase, Lilacs via the Virtual Health Library, Scopus, and Web of Science. To ensure comprehensive literature coverage, the reference lists of studies included and relevant systematic or literature reviews identified through the search were also reviewed. The bibliographic search was performed in November 2023, with an update in March 2024.

The implemented search strategies are presented in Multimedia Appendix 1. The search terms were defined based on the population, intervention, and outcomes, following the PICOS structure. These terms include controlled vocabulary specific to each database, as well as uncontrolled vocabulary terms, synonyms, natural language, related terms, expressions, and truncations, all aimed at broadening the scope of the search. The strategies were developed in collaboration with a librarian experienced in conducting systematic review searches. Initially, the MEDLINE strategy was created with input from the research team and subsequently reviewed by a specialist with expertise in the research topic. Once the MEDLINE strategy was finalized, it was adapted to the syntax and subject headings of the other databases.

The literature search results were uploaded to Rayyan, where duplicates were eliminated, and an initial screening of abstracts and titles was conducted to identify potential studies for inclusion. Finally, the full texts of these potential studies were assessed to determine their eligibility. Adhering to PRISMA recommendations, both steps were performed independently by 2 reviewers [ACMS and LESL]. Any disagreements not resolved through discussion between the reviewers were arbitrated by a third reviewer [BZO]. The complete list of articles excluded during the full-text assessment is detailed in Multimedia Appendix 2, along with the documented reasons for study exclusions.

### Data Collection Process and Data Items

Studies that passed the eligibility stage proceeded to data extraction, which was performed using a predefined Excel spreadsheet by the same independent reviewers [ACMS and LESL]. The following information was extracted from each study: title, year of publication, authors, corresponding author contact, language, abstract, objectives, population (inclusion criteria, size, and characteristics), methods (type of study, time horizon, study site, comparators, and statistical significance), outcome measures (policies implemented, including health care improvements and preventive measures, hospitalization rates or duration, and mortality), funding, and conflicts of interest. Regarding the registries, the information collected included the type of registry (whether it was an injury or trauma registry), registry age, and specific focus of the registry. When disagreements arose, the full-text articles were reviewed again by the reviewers.

### Data Synthesis

Summaries of study characteristics were presented in a table, and data synthesis was explored based on outcomes. The policy changes and designs, including preventive measures for injuries, health care improvements, surveillance actions, stakeholder partnerships, among others, were also synthesized in a table. For each hospitalization rate or duration, and for mortality, the intervention results were reported through number or percentage differences. A meta-analysis could not be conducted due to considerable methodological heterogeneity among the included studies and the small number of studies reporting the same outcome; consequently, publication bias could also not be assessed.

### Quality Assessment

Two reviewers [ACMS and LESL] independently assessed the methodological quality of studies using the Newcastle-Ottawa Scale (NOS) [27] for cohort studies and an adaptation of this tool for cross-sectional studies [28]. This tool was deemed the most appropriate considering the study designs of interest in this review. According to the instructions of the NOS tool, the assessment was conducted considering the study as a whole, regardless of the specific outcomes analyzed in this systematic review.

The NOS is organized around 3 main dimensions of observational studies: participant selection, comparability between groups, and outcome evaluation. Quality assessment in NOS uses a star system. For cohort studies, up to 9 stars can be awarded across the 3 domains: selection (up to 4 stars), comparability (up to 2 stars), and outcome (up to 3 stars) [27]. For the adapted tool for cross-sectional studies, up to 10 stars can be awarded across 3 domains: selection (up to 5 stars), comparability (up to 2 stars), and outcome (up to 3 stars) [28].

To standardize study evaluation, specific criteria were defined for all studies. In the selection dimension, the item “Representativeness of the exposed cohort” considered the country of the registry to assess population representativeness. For the item “Was follow-up long enough for outcomes to occur” in the outcome dimension, studies were awarded a star if they had a minimum follow-up period of 3 years. The bias assessment involved independent evaluation by pairs (ACM-d-S and LESL), with discrepancies resolved by a third reviewer (TFGdSB).

## Results

### Study Selection

**Figure 1.**
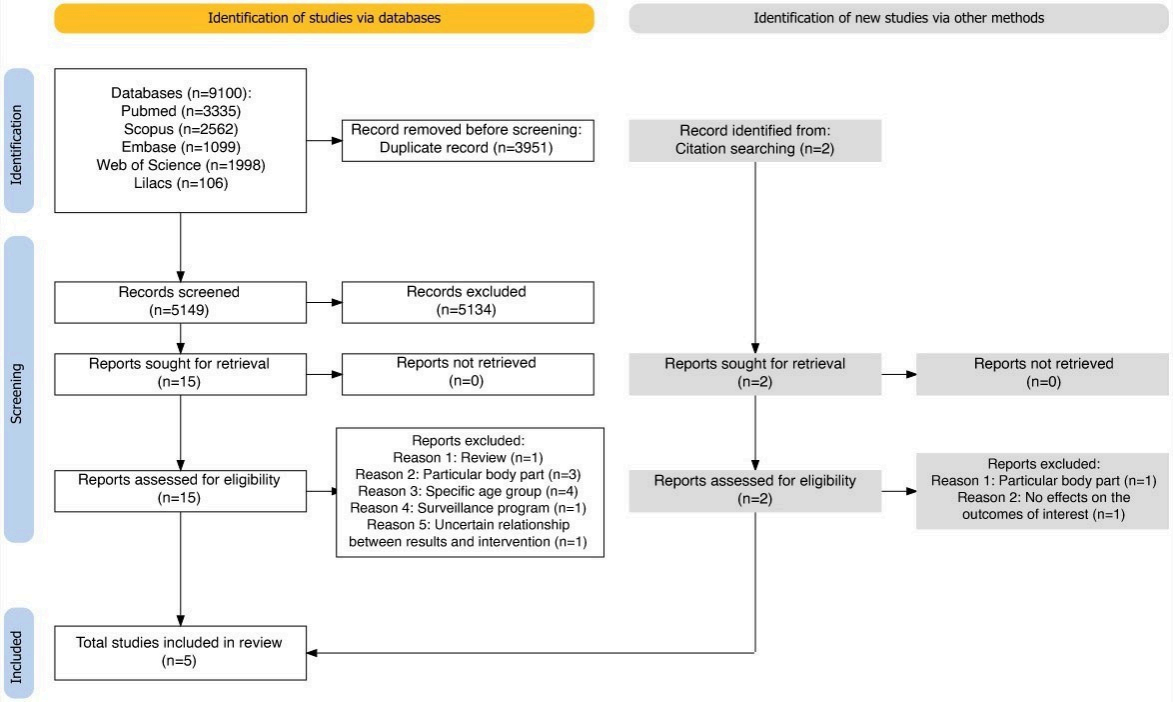
Flow diagram of the search and selection process of the studies included in the review.

The database searches yielded 9100 potentially relevant records. After removing duplicates, 5149 unique records underwent screening based on their titles and abstracts. Subsequently, 15 full-text documents were examined, resulting in the inclusion of 5 papers [29-33]. In addition, a search was conducted in the references of the initial studies included. However, no additional articles meeting the inclusion criteria were identified through these searches (Figure 1).

A total of 12 studies were excluded from our review [34-45], with reasons for exclusion listed in Checklist 2. Among these exclusions, 1 study was disregarded due to its nature as a literature review [34]. In addition, 8 focused exclusively on a particular body part [38-41] or age group [36,42-44]. In one of them, the relationship between the reported results and the registry was not clear [35]. Another showed no associations on the outcomes of interest for this review [37]. Finally, 1 study presented results that appeared to extend beyond the implementation and use of data from registries; it reported findings from its surveillance program [45].

### Study Characteristics

The characteristics of each study included are provided in Tables2  3. Of the 5 studies, 3 conducted in Germany involved data from trauma registries, with publication years ranging from 2004 to 2013 [29-31]. The remaining 2, one conducted in Cardiff, Wales, published in 2004 [32], and another in Harstad and Trondheim, Norway, published in 1995 [33], used data from injury registries.

Among the studies included, 4 used an observational cohort design, using a before-and-after approach [29-32]. One of these included a corresponding comparison group [29], while 2 others compared the obtained results with expected values as well [30,31]. Notably, 1 adopted a mixed design, incorporating a cross-sectional assessment involving hospitals to evaluate their efforts to improve care quality [31]. The fifth study classified itself as quasi-experimental; however, the outcome of interest for this review was assessed using a cohort [33].

Four of the studies described policymaking outcomes. Two, focusing on trauma registries, detailed health care improvements [29,31]. Meanwhile, 2 other centered on injury registries presented results on diverse approaches to injury management, including preventive measures against assault [32] and traffic injuries [33]. In addition, 2 studies reported associations of these policies on hospitalization [31,33], and 4 described the mortality outcomes [29-31,33].

**Table 2.** Characteristics of studies included.

First author, year (country)	Title	Registry type	Described outcomes
Helm M, 2013 (Germany) [29]	The Trauma Register DGU as the basis of medical quality management. Ten years’ experience of a national trauma center exemplified by emergency room treatment	Trauma	Policymaking and mortality
Ruchholtz S, 2008 (Germany) [30]	Reduction in mortality of severely injured patients in Germany	Trauma	Mortality
Ruchholtz S, 2004 (Germany) [31]	External quality management in the clinical treatment of severely injured patients	Trauma	Policymaking, hospitalization, and mortality
Warburton AL, 2004 (Wales) [32]	Development, usage, and importance of accident and emergency department–derived assault data in violence management	Injury	Policymaking
Ytterstad B, 1995 (Norway) [33]	The Harstad Injury Prevention Study: evaluation of hospital-based injury recording and community-based intervention for traffic injury prevention	Injury	Policymaking, hospitalization, and mortality

**Table 3.** Details of studies included.

First author, year (country)	Study details				
	Study type	Time horizon	Population	Comparator	Funding
Helm M, 2013 (Germany) [29]	Observational (cohort)	Almost 10 years, divided into 4 periods: January 1998-December 1998; January 1999-December 2000; January 2001-December 2005; January 2006- March 2007.	Patients admitted via emergency rooms at a Level I trauma center (all patients: n=2239, and ISS^a^ ≥16: n=1174)	First observed period and TR-DGU^b^ hospitals data (ISS ≥16, national trauma centers from Germany)	Not explicitly indicated in the study
Ruchholtz S, 2008 (Germany) [30]	Observational (cohort)	7 years, from 1999 to 2005	Patients with ISS ≥16 admitted to 105 hospitals of the TR-DGU (n=11,013)	First observed period and values expected	not explicitly indicated in the study
Ruchholtz S, 2004 (Germany) [31]	Observational (cross-sectional+ cohort)	3 years, from 1999 to 2001	Hospitals transmitting data for ≥100 patients to TR-DGU (n=21) and patients with ISS ≥16, directly transferred from the accident site (n=2702)	First observed period and values expected	German Research Foundation and German Society for Trauma Surgery
Warburton AL, 2004 (Wales) [32]	Observational (cohort)	7 years, from April 1, 1995, to March 31, 2002, encompassing each annual period from April 1 to March 31.	All assault patients treated at the Cardiff Accident and Emergency (A&E) department	No comparator	Wales Office of Research and Development (WORD).
Ytterstad B, 1995 (Norway) [33]	Quasi-experimental+ cohort	7 years, from July 1, 1985, to June 30, 1993, was divided into three periods of 30 months each.	Individuals treated for traffic accident injuries in hospitals in two Norwegian cities, Harstad (n=22,970) and Trondheim (n=158,911).	Trondheim and first observed period	Norwegian Research Council for Science and the Humanities (NAVF) and Norwegian Directorate of Health/The Royal Norwegian Ministry of Social Affairs

a ISS: Injury Severity Score.

b TR-DGU: Trauma Register of the German Society of Trauma Surgery.

### Outcomes

#### Policymaking

In total, 4 of the 5 studies included demonstrated the use of injury or trauma registry data as a policymaking tool. The key policies identified, including health care improvements and preventive measures, are synthesized in Table 4.

Two studies conducted in Germany involved data from trauma registries and demonstrated their use in continuously improving the care of hospitalized patients. One of these [29] shows the evolution of emergency room management in a national trauma center over a decade, marked by significant improvements across four distinct phases. These improvements resulted from ongoing monitoring of hospital data and comparisons with the Trauma Register of the German Society of Trauma Surgery (TR-DGU) to identify areas needing enhancement. Interventions included incorporating diagnostic exams and imaging procedures in the emergency room, refining personnel training protocols, and implementing time-efficient treatment algorithms. The other study [31] involved interviews with 21 hospitals regarding their efforts to improve care quality based on trauma registry data feedback. Optimization measures were introduced in the emergency room, surgical care, and intensive care. In the emergency room, changes included the installation of digital X-ray systems and spiral CT scanners. Organizational improvements focused on enhancing interdisciplinary coordination through quality circles and guidelines, as well as training and documentation. In surgical and intensive care, optimization targeted therapy areas, although specific details were not provided.

The 2 studies that described policymaking results related to injury registries presented preventive initiatives against assault and traffic injuries. For violence prevention, 1 study [32] detailed efforts by a multisector steering group, including assault awareness campaigns, targeted policing initiatives, and night transport management strategies. The use of data to guide judicial decisions, particularly for alcohol and entertainment license applications, was also highlighted. The authors concluded that using injury data from assault patients attending A&E departments in Cardiff, Wales, effectively targeted police and local resources to reduce city-wide violence. The other study [33] also focused on traffic injury prevention through a multisector group’s efforts. It described education initiatives targeting high-risk groups, traffic safety measures like installing stoplights and constructing separate pedestrian and cyclist roads, and enhanced traffic enforcement such as vehicle checks and speed limit enforcement. Legislative changes included assigning local health authorities the responsibility for accident prevention. The study emphasized using data to inform various stakeholders about traffic injury risks and prevention strategies. Conducted in Harstad, Norway, the data were actively used in a community-based injury prevention program.

**Table 4.** Key policies identified in studies.^a^

Policies	Studies
Trauma registry	
Continuous quality management in health care	3 [29,31]
Changes in emergency room management	2 [29,31]
Introducing exams and imaging diagnosis in the emergency room	2 [29,31]
Adjusting training and documentation	2 [29,31]
Other measures (eg, introducing therapy in the emergency room)	2 [29,31]
Changes in intensive care (organization, diagnosis, and therapeutic measures)	1 [31]
Changes in surgical care (organization, diagnosis, and therapeutic measures)	1 [31]
Injury registry	
Violence prevention by multiple sectors group	1 [32]
Assault awareness campaigns (eg, in schools and public libraries, and for bar and club managers)	1 [32]
Targeted policing initiatives (eg, a task force to hotspot areas, and targeting alcohol-related street crime)	1 [32]
Night transport management (eg, meetings to identify barriers and opportunities for education and staff protection)	1 [32]
Use of data to guide judicial decisions (eg, alcohol and entertainment license applications)	1 [32]
Traffic injury prevention by multiple sectors group	1 [33]
Traffic injury education initiatives and campaigns to high-risk groups (eg, in schools and driving schools, at health fairs and shopping centers, for parents of small children, for police and automobile societies, and through media campaigns)	1 [33]
Traffic safety measures (eg, installation of additional stoplights, constructing separate pedestrian and cyclist roads, planning safe pathways for children going to school, and loaning safety chairs for small children)	1 [33]
Enhanced traffic enforcement (eg, checks on vehicles, speed limit enforcement, and curfews for serving alcohol in bars and restaurants)	1 [33]
Legislative change (eg, a national law assigning local community health authorities’ responsibility for accident injury prevention)	1 [33]
Use of data to inform educational institutions, city planners, private and public organizations, and the community	1 [33]

a Including health care improvements and preventive measures, among others.

#### Hospitalization

Although a meta-analysis for hospitalization results was planned, it could not be conducted due to the considerable methodological heterogeneity among the studies included and the small number of studies reporting this outcome (n=2). Since quantitative synthesis is not appropriate, the results observed by the authors are presented in narrative form below. Relevant findings identified in the studies can be found in Table 5.

**Table 5.** Hospitalization and mortality results identified in studies.

Outcome or studies	Follow-up	Results
Hospitalization		
Ruchholtz S, 2004 (Germany) [31]	1999‐2001	3 days in the average length of stay in the ICU^a^ among patients with an ISS^b^ ≥16 (from 16 to 13 days, *P*<.05)
Ytterstad B, 1995 (Norway) [33]	1987‐1992	42% per year reduction in admissions from traffic injuries (from 45 to 16 admissions)
Mortality		
Helm M, 2013 (Germany) [29]	1998‐2007	6.0%^c^ in mortality among all patients (from 10.8% to 4.8%, *P*<.05) 20.1%^c^ in mortality among patients with an ISS ≥16 (from 33.3% to 13.2%, *P*<.05) 11.3%^c^ greater reduction in mortality among ISS ≥16 patients compared with TR-DGU hospitals (−20.1% vs −8.8%)
Ruchholtz S, 2008 (Germany) [30]	1999‐2005	4.1%^c^ in observed mortality among patients with an ISS ≥16 (from 22.8% to 18.7%) 3.4%^c^ in expected mortality among patients with an ISS ≥16 (18.7% vs 22.1%)
Ruchholtz S, 2004 (Germany) [31]	1999‐2001	4.0% in expected mortality among patients with an ISS ≥16 (17.5% vs 21.5%)
Ytterstad B, 1995 (Norway) [33]	1985‐1993	66.7%^c^ reduction in traffic-related deaths (from 6 to 2, *P*<.12)

a ICU: intensive care unit.

b ISS: Injury Severity Score.

c Values calculated by the systematic review authors based on study data.

Two studies described hospitalization results: 1 presented findings related to the implementation and use of a trauma registry, and the other presented results related to an injury registry. The first study [31], conducted in Germany, used continuous monitoring of hospital data to introduce optimization measures in the emergency room, surgical care, and intensive care. It compared the change in average values for the length of stay in the intensive care unit and total hospital stay over 3 years (1999‐2001), involving data from 2702 patients with an Injury Severity Score (ISS)≥16 who were directly transferred from the accident site. The authors found no significant reduction in the total average length of hospital stay for patients (from 29 to 28 days). However, there was a significant reduction of 3 days in the average length of stay in the intensive care unit (from 16 to 13 days, *P*<.05).

In the second study [33], the injury registry was actively used in a community-based traffic injury prevention program conducted in Norway. The authors observed hospitalization results by comparing the 1987 sample of all admissions from traffic accidents at Harstad Hospital with the mean annual admissions during 1990‐1992. As a result, a 42% per year reduction in admissions from traffic injuries was observed, decreasing from an average of 45 admissions per year to 26.

#### Mortality

The planned meta-analysis for the mortality could also not be conducted due to the considerable heterogeneity in terms of design and methods among the studies included that reported this outcome (n=4). Therefore, a quantitative synthesis was deemed inappropriate, and the findings from the studies are summarized in the text below. Relevant results identified in the studies can be found in Table 5.

Regarding the studies that focused on trauma registries, all were conducted in Germany and observed results on mortality. One study [29] implemented various interventions in the emergency room of a national trauma center through continuous monitoring of hospital data, including the refinement of personnel training protocols and the incorporation of diagnostic exams and imaging procedures. This study observed a significant reduction in hospital mortality among all patients, decreasing from 10.8% to 4.8% (−6.0%, *P*<.05), and specifically among patients with an ISS ≥16, decreasing from 33.3% to 13.2% (−20.1%, *P*<.05). The total observation period covered almost 10 years (January 1998 to March 2007), during which a total of 2239 trauma patients were admitted via the emergency room, including 1174 patients with ISS ≥16. Similar reductions were observed in hospitals part of the TR-DGU, where mortality rates among patients with an ISS ≥16 decreased by 8.8%, from 24.9% to 16.1%. However, the reduction in the studied hospital was notably more substantial, with an 11.3% greater decrease (−20.1% vs −8.8%).

Another study [30] evaluated patient data starting from 1999, which marked the beginning of receiving annual feedback in the form of reports comparing hospital data with TR-DGU results to identify areas for improvement and support continuous quality management. This study demonstrated that consistent feedback of treatment data to hospitals is associated with positive changes in survival prognosis. During the observation period (1999 to 2005), data from a total of 11,013 severely injured patients (ISS ≥16) in 105 hospitals were documented. Mortality significantly decreased by 4.1%, from 22.8% in 1999 to 18.7% in 2005, compared with an expected rate of 22.1% for that year, which represents a 3.4% reduction in expected mortality. The same author [31] had already noted that the observed mortality rate was lower than the expected rate (−4.0%, 17.5% vs 21.5%) after the introduction of optimization measures in the emergency room, surgical care, and intensive care in 21 hospitals that transmitted data to TR-DGU between 1999 and 2001. Despite reporting potential limitations due to the expected mortality calculation method being based on United States data and finding no significant change in overall mortality rates (16.6% vs 17.5%).

The Norway study [33] aimed to test the feasibility of an injury registry and evaluate its results within a community-based traffic injury prevention program over a follow-up period of 7.5 years, from July 1, 1985, to June 30, 1993, divided into 3 periods of 30 months each. The study observed a decrease in the number of traffic-related fatalities in Harstad, with a total of 10 resident deaths: 6 in period 1, 2 in period 2, and 2 in period 3 (*P*<.12). This represents a 66.7% reduction in traffic-related deaths from period 1 to period 3.

### Quality Assessment

Among the 5 studies included in this review, 4 were rated as good quality [29-31,33], while only 1 study was assessed as fair quality [32]. The quality assessment results for the cohort studies are summarized in Multimedia Appendix 3, and the results for the cross-sectional studies are presented in Multimedia Appendix 4.

The only study classified as fair received just 2 stars in the assessment of the dimension of selection for exposed and unexposed cohorts. It was deemed not representative of the general population, specifically the country where the registry is located, as outlined in the methodology of this review. In addition, it lacked comparability of results with an unexposed cohort. While the NOS assigns higher scores to cohort studies that include nonexposed groups, similar to the other 2 studies in this review [30,31], this study did not aim to compare its results with a nonexposed cohort, as it was designed as a before-and-after study.

The analysis of evidence certainty using the Grading of Recommendations Assessment, Development and Evaluation (GRADE) tool was not conducted due to the designs and objectives of the studies. The diverse methodologies used, along with their focus on practical implications rather than outcomes as such, limited the applicability of GRADE.

## Discussion

### Principal Findings

The findings of this review suggest that injury and trauma registries have the potential to inform policymaking, including preventive measures, health care improvements, and surveillance actions, which can lead to enhanced health outcomes. The studies indicate that trauma registries are primarily designed to provide information that can enhance the efficiency and quality of care for hospitalized patients, with findings including reductions in intensive care unit stay duration and the number of deaths [29-31]. Meanwhile, injury registries are used in community-based injury prevention to identify at-risk populations and health needs, as well as to plan and implement prevention policies that address local priorities [32,33]. One of the 2 studies on injury registries demonstrated reductions in traffic-related hospital admissions and deaths [33]. The other, while not reporting a decrease in these outcomes, noted a reduction in the overall number of assault cases attending the emergency room in the sixth and seventh years of the registry [32]. These studies also emphasized the importance of involving multiple sectors, beyond just health, in injury prevention efforts. All studies highlighted the value of registries for scientific research.

However, it is important to note that the vast majority of studies retrieved from our search used registry data to generate scientific knowledge rather than to assess the impact or effects of the registry itself. For instance, 1 study aimed to identify predictors of prehospital versus hospital mortality due to road traffic injuries in an Iranian population, using data from an integrated road traffic injury registry [46]. Another study examined the epidemiology of occupational injury hospitalizations in the United Arab Emirates using a trauma registry [47]. While some studies evaluated the results of implemented policies and measures, such as one that presented significant trends and developments in acute trauma care over 20 years [35], they did not intend or did not establish causality related to the implementation and use of registry data. Consequently, despite the large number of studies identified in our search, only a few advanced to the full reading phase, as many were excluded during the screening stage after reviewing their titles and abstracts.

An important observation from this review is the notable scarcity of studies dedicated to exploring the effects or results of the use of registry data on injury outcomes. In addition, a crucial point to highlight when interpreting the results is that even the few studies included in the review reflect correlations rather than causalities. This is particularly important as the studies did not adopt designs that allowed for the control of external factors, making it impossible to conclude that the observed results can be attributed solely to the implementation and use of injury or trauma registry data. Currently, there are no impact publications.

### Limitations

The limitations of our review include the cautious search strategy, which may have excluded studies assessing surveillance information systems or other types that capture injury data, or that collected data solely for that specific research project. We were particularly careful not to subjectively define which studies originated from injury registries. Therefore, we focused on studies that explicitly identified their data sources as such. Our conservative approach focused on the terms “injury registry” and “trauma registry” to ensure the inclusion of studies containing structured registry data with direct relevance to our research question. For example, a study evaluating the impact of the Life in Traffic Project—implemented in 2010 across various Brazilian capitals to reduce traffic-related deaths and serious injuries, which is based on an integrated analysis of data from multiple sectors involved in road traffic injury issues—was not included in our systematic review [48]. The study used interrupted time series models to compare mortality rates before and after the project’s implementation, revealing positive outcomes in some cities but concerning increases in others. It highlights the necessity of ongoing evaluations through data registries to enhance public policy analysis and monitoring. Despite its relevance, the tool implemented by this project did not function as an injury or trauma registry. This limitation has prompted the Brazilian Ministry of Health to organize a workshop aimed at establishing a national concept for accident and violence registries, which took place in October 2024.

Although the NOS was considered the most appropriate tool for analyzing the methodological quality of the studies included in this review, we still identified limitations in its application. Most of the studies included did not aim to compare their results with a nonexposed cohort, as they were designed as before-and-after studies. This led to one of them being classified as “fair” because the NOS assigns higher scores to cohort studies that include nonexposed groups. In addition, we had to use an adapted version of the tool for cross-sectional studies, and an analysis of evidence certainty using the GRADE tool was not conducted due to the designs and objectives of the studies. Furthermore, it is crucial to acknowledge the potential influence of publication bias, where studies yielding positive outcomes are more likely to be published, thereby shaping the landscape of the literature.

The studies included collectively provided insights into the results of trauma and injury registries on reducing hospitalizations and mortality; however, variations in methodology and context limited the ability to draw synthesized results through meta-analysis.

### Comparison With Previous Work

To our knowledge, this is the first systematic review aimed at mapping the effects and results related to registry data, including those that encompass all types of injuries, regardless of their severity. Previous research has predominantly focused on this relationship with trauma systems, as evidenced by a meta-analysis that demonstrated a 15% reduction in mortality in favor of the presence of a trauma system [22], as well as by another systematic review that sought to identify which components contributed to its effectiveness [21]. In addition, we can mention a study that exclusively addressed the most common functions of trauma registry data in the formulation of health policies but did not examine its effects [13].

### Conclusions

This study is especially timely, given the current efforts to develop an injury registry in Brazil. It addressed the existing knowledge gap related to the results of injury and trauma registries on policymaking and injury outcomes. Highlighting our acknowledgment that the registry alone does not directly influence morbidity and mortality; rather, these outcomes are indirect results of evidence-based policies and preventive measures implemented [13,34,49]. Besides, we emphasize that the findings of the studies included represent associations, making it essential to temper expectations regarding the causalities of their effects, as well as to exercise caution in the transportability of results from European studies to the Brazilian context.

Factors such as distinct social, economic, and cultural characteristics, access to health care, trauma burden, practices related to injury management and policies, along with the handling and use of data from the implemented injury registry, may influence the outcomes observed in studies conducted in Europe. Public health decision-makers should consider the differences in the distribution of potential effect modifiers when interpreting the results and their applicability in Brazil. Some studies discuss limitations and adjustments that can be applied through the combination of assumptions and statistical approaches to ensure the unbiased transfer of results obtained from different populations [50,51].

Furthermore, the tool being developed is expected to be low-cost, primarily based on the unification and sharing of existing information systems that currently lack interoperability. This approach represents one of the solutions identified in a study published in 2018 to address the challenges of implementing trauma registries in low- and middle-income countries [49]. Another study highlighted that trauma registries are rarely found in mid to low-income countries due to the lack of digital resources needed to maintain a costly registry. According to the author, until 2017, many countries with a significant trauma burden—including India, South Africa, Spain, China, and Brazil—had yet to develop nationwide trauma registries [52]. The national injury registry currently in development for Brazil aims not only to reduce costs but also to enhance the quality of data and analysis. The main objective is to strengthen epidemiological surveillance to support policies and preventive measures against accidents and violence.

In conclusion, the establishment of an injury registry in Brazil presents a significant opportunity to improve health outcomes through informed policymaking. While it is crucial to set appropriate expectations regarding its effects on morbidity and mortality, its role in facilitating preventive measures and enhancing surveillance capabilities remains valuable.

Future reviews should aim to broaden the scope of inquiry to include studies derived from other data sources, even if they were not explicitly identified as injury or trauma registries, but which still align with the concept of accident and violence registries defined by Brazil after our study. In addition, it would be beneficial to conduct primary studies that examine the effects of these registries on the quality of surveillance. Without neglecting to mention that it is essential to apply methodologies that allow for the evaluation of the causal impact on health outcomes, particularly in the Brazilian context.

## Supplementary material

10.2196/67115 Multimedia Appendix 1 Search strategy.

10.2196/67115 Multimedia Appendix 2 Full-text evaluated and studies excluded.

10.2196/67115 Multimedia Appendix 3 Newcastle-Ottawa Scale quality assessment for cohort studies.

10.2196/67115 Multimedia Appendix 4 Newcastle-Ottawa Scale quality assessment for cross-sectional studies.

10.2196/67115 Checklist 1 PRISMA 2020 main checklist.

10.2196/67115 Checklist 2 PRISMA abstract checklist.

## Abbreviations

GRADE: Grading of Recommendations Assessment, Development and Evaluation
ISS: Injury Severity Score
NOS: Newcastle-Ottawa Scale
PICOS: population, intervention, comparison, outcomes, and study
PRISMA: Preferred Reporting Items for Systematic Reviews and Meta-Analyses
PROADI-SUS: Programa de Apoio ao Desenvolvimento Institucional do Sistema Único de Saúde
PROSPERO: Prospective Register of Systematic Reviews
TR-DGU: Trauma Register of the German Society of Trauma Surgery
WHO: World Health Organization
