# Supplementary material for: Effects of Injury Registry Data on Policymaking, Hospitalizations, and Mortality: Systematic Review
Source: JMIR Public Health Surveill. 2025 Sep 10;11:e67115. doi: 10.2196/67115 (PMC12422531; doi:10.2196/67115)
Supplement: Multimedia Appendix 2 [file publichealth-v11-e67115-s002.docx]

Full-text evaluated and studies excluded.

| **First author, year (country)** | **Title** | **Reason for exclusion** |
| --- | --- | --- |
| Bouillon B, 2016 (Germany) [34] | Treatment of severely injured patients: Impact of the German Trauma Registry DGU® | Review |
| -, 2014 (Germany) [35] | 20 years of trauma documentation in Germany--actual trends and developments | Uncertain relationship between results and intervention |
| Holstein JH, 2016 (Germany) [38] | Influence of the pelvic trauma registry of the DGU on treatment of pelvic ring fractures | Particular body part: pelvic ring |
| Hauschild O, 2008 (Germany) [39] | Mortality in patients with pelvic fractures: results from the German pelvic injury register | Particular body part: pelvic ring |
| Richard-Denis A, 2021 (Canada) [40] | An evaluation of the representativeness of a national spinal cord injury registry: a population-based cohort study | Particular body part: spinal cord |
| Noonan VK, 2012 (Canada) [41] | The Rick Hansen Spinal Cord Injury Registry (RHSCIR): a national patient-registry | Particular body part: spinal cord |
| Pressley JC, 2005 (USA) [42] | A national program for injury prevention in children and adolescents: the injury free coalition for kids | Specific age group:  under 17 years old |
| Ytterstad B, 1996 (Norway) [43] | The Harstad injury prevention study: community-based prevention of fall-fractures in the elderly evaluated by means of a hospital-based injury recording system in Norway. | Specific age group:  65 years or more |
| Ytterstad B, 1998 (Norway) [44] | Harstad injury prevention study: prevention of burns in young children by community-based intervention | Specific age group:  under 5 years old |
| Razzak JA, 2012 (Pakistan) [45] | A successful model of Road Traffic Injury surveillance in a developing country: process and lessons learnt | Surveillance program |
| Weiner EJ, 2009 (USA) [36] | Application of electronic surveillance and global information system mapping to track the epidemiology of pediatric pedestrian injury | Specific age group:  under 18 years old |
| Isles S, 2017 (New Zealand) [37] | The New Zealand Major Trauma Registry: the foundation for a data-driven approach in a contemporary trauma system. | No effects on the outcomes of interest |
