# Supplementary material for: Effects of Injury Registry Data on Policymaking, Hospitalizations, and Mortality: Systematic Review
Source: JMIR Public Health Surveill. 2025 Sep 10;11:e67115. doi: 10.2196/67115 (PMC12422531; doi:10.2196/67115)
Supplement: Multimedia Appendix 3 [file publichealth-v11-e67115-s003.docx]

NOS quality assessment for cohort studies.

| **Cohort studies**  **(n = 5)** | **Selection** | | | | **Comparability** | **Outcome** | | | **Total Score^a^** |
| --- | --- | --- | --- | --- | --- | --- | --- | --- | --- |
|  | Representativeness of the exposed cohort | Selection of the non-exposed cohort | Ascertainment of exposure | Demonstration that outcome of interest was not present at start of study | Comparability of cohorts on the basis of the design or analysis | Assessment of outcome | Was follow-up long enough for outcomes to occur | Adequacy of follow up of cohorts |  |
| Helm M, 2013 (Germany) [29] | - | ★ | ★ | ★ | ★★ | ★ | ★ | ★ | Good |
| Ruchholtz S, 2008 (Germany) [30] | ★ | - | ★ | ★ | ★★ | ★ | ★ | ★ | Good |
| Ruchholtz S, 2004 (Germany) [31] | ★ | - | ★ | ★ | ★★ | ★ | ★ | ★ | Good |
| Warburton AL, 2004 (Wales) [32] | - | - | ★ | ★ | ★★ | ★ | ★ | ★ | Fair |
| Ytterstad B, 1995 (Norway) [33] | - | ★ | ★ | ★ | ★★ | ★ | ★ | ★ | Good |

^a^ Total Score: Scores were categorized as good (3-4 stars in selection, 1-2 stars in comparability, and 2-3 stars in outcome), fair (2 stars in selection, 1-2 stars in comparability, and 2-3 stars in outcome), or poor (0-1 star in selection, or 0 stars in comparability, or 0-1 star in outcome).
