# Supplementary material for: Effects of Injury Registry Data on Policymaking, Hospitalizations, and Mortality: Systematic Review
Source: JMIR Public Health Surveill. 2025 Sep 10;11:e67115. doi: 10.2196/67115 (PMC12422531; doi:10.2196/67115)
Supplement: Multimedia Appendix 4 [file publichealth-v11-e67115-s004.docx]

NOS quality assessment for cross-sectional studies

| **Cross-sectional studies**  **(n = 1)** | **Selection** | | | | **Comparability** | **Outcome** | | **Total Score^a^** |
| --- | --- | --- | --- | --- | --- | --- | --- | --- |
|  | Representativeness of sample | Sample size | Non-respondents | Ascertainment of the exposure (risk factor) | The subjects in different outcome groups are comparable | Assessment of outcome | Statistical test |  |
| Ruchholtz S, 2004 (Germany) [31] | ★ | ★ | ★ | - | ★ | ★ | ★ | Good |

^a^ Total Score: Scores were categorized as good (3-4 stars in selection, 1-2 stars in comparability, and 2-3 stars in outcome), fair (2 stars in selection, 1-2 stars in comparability, and 2-3 stars in outcome), or poor (0-1 star in selection, or 0 stars in comparability, or 0-1 star in outcome).
